# A Case of Supposed Periostitis in the Neighborhood of the Foramen Magnum

**Published:** 1872-12

**Authors:** Frederick P. Henry

**Affiliations:** Philadelphia


### A CASE OF SUPPOSED PERIOSTITIS IN THE NEIGHBORHOOD OF THE FORAMEN MAGNUM.

BY FREDERICK P. HENRY, M.D., Philadelphia.

The principal, though not the only point of interest in the following case is the fact that the study of the mechanism of one of our commonest movements—that of nodding the head—was the only means by which the seat of the disorder could be ascertained.

T. H., æt. 30, driver of a street car, came to the Catherine Street Dispensary on the 30th of January, 1872, complaining of a tingling sensation in both hands and arms, "as if they were asleep;" also of a feeling of weakness and numbness in both upper extremities. The tingling is not constant, but is at once produced by taking hold of anthing, whether gently or with force. For example, in turning the brake of his car he feels it; he also feels it when he takes out his watch. He can produce it at any time by slightly bending his head forward, as in the act of nodding. This act, by the way, is performed when he looks at his watch, and probably is when he turns his brake; so that it is extremely doubtful whether, instead of ascribing the abnormal sensation to the contact of the foreign bodies, he should not have ascribed them to the accompanying motion of the head.

Occasionally he has momentary attacks of the vertigo and dimness of sight, during which his face feels very hot. His appearance is that of a man in exuberant health. He stands erect and firmly on his legs. He has a fine color—too much color, one would say *after hearing the symptoms*—although it seems to have been habitual. His appetite is good ; he sleeps well ; he is neither fat nor lean, but well nourished, and, in fact, were it not for the symptoms detailed above, his physical condition would leave nothing to be desired.

On first seeing the patient, I had little time to bestow upon his case, but, perceiving it to be one of some interest, I determined not to lose sight of him.

I prescribed ten grains of the bromide of potassium three times a day, and two compound cathartic pills for three nights on going to bed.

February 5.—He has taken his medicine regularly, but the symptoms remain the same ; he thinks, however, that he feels decidedly weaker. I prescribed iron and quinine—somewhat empirically, it must be acknowledged—ordered him to attend to the state of his bowels, and to take a hot foot-bath every night on going to bed.

February 13.—At this interview I examined the patient more carefully as to his previous history than I had done hitherto. I ascertained that in the month of June, 1871, he had a chancre situated upon the frænum præputii, followed by three non-supperating buboes, two of which were situated in the right groin and one in the left. He received no constitutional treatment, but, to use his own words, he "burnt the chancre, and it soon got well." He has had no eruption upon the body, to his knowledge, no sore throat, and no rheumatic pains. His throat, on examination, appears perfectly healthy. There is an enlarged and indurated gland upon the back of the neck, to the left of the median line and about one inch from the extremity of the left mastoid pracess.

At the last October elections (1871) he was wounded with a black-jack over the occipital bone; the scar is situated directly upon the nucha, and extends about an inch below it. After receiving the wound, he remained insensible for three hours. He feels stronger than when last seen, but the tingling sensation is still produced in all the fingers of both hands by slightly bending the head forward.

I made an ophthalmoscopic examination of the right eye, and found nothing abnormal. In the left eye there is an anterior synechia, caused by a prolapse of the iris from a corneal wound, giving the pupil a transversely elliptical shape. I did not examine the left eye, at the patient's request; he stated that it was "weak." I advised him to begin the use of iodide of potassium; but he thought he had derived some benefit from the last prescription, a small quantity of which still remained, and wished to finish it, to which I agreed.

February 20.—About the same. If there is any change it is for the worse. By careful examination I ascertained that the tingling occurs during the first stage of the act of nodding, viz: during the movement of the occipital bone upon the atlas. When the act proceeds farther, the tingling is increased. Ordered gr. x of potass. iodid. three times a day.

At this point a few words upon the act of nodding will not be out of place. This act is the product of a combined movement of the occipital bone upon the atlas, and of all the seven cervical vertebræ upon each other. It may, for our present purpose, be divided into two stages, the first stage consisting of the movement of the occipital bone upon the atlas, the second, of that of the cervical vertebræ upon each other. Let any one, preserving the cervical portion of the spine rigid, attempt to bend his head forward, and he will find that the motion—which is that of the occipital bone upon the atlas—is very limited. In order to the full performance of the act of nodding, it is necessary that the whole cervical portion of the spine participate in it. When this is done, the slight anterior convexity of the cervical region is abolished, and, the movement being carried still further, a slight concavity substituted, while the convexity is transferred to that portion of the spinal column which covers and protects the posterior columns of the cord.* The spine of the vertebra prominens, as well as the other cervical spine, becomes more prominent, as any one may observe by placing the hand firmly upon a cervical spine, and bending the head forward.

In an article on "fractures of the odontoid process," by Dr. Stephen Smith, of New York; (*American Journal of the Medi-*

*The mechanism of this (nodding) was tested upon a recent section of the cranium and cervical vertebral region.

*cal Sciences, for October*, 1871) a number of cases of that accident are detailed. In Case IV., in which there was caries of the atlas and axis, "pricking sensations were felt in the right superior extremity, and this was evidently a loss of muscular power in it." In Case V. there was "fracture of the axis in such a manner as to include the base of the process." It was "not till sixteen months after the accident" that the patient "complained of numbness and want of power in the left arm."

Now, in the case of T. H. a violent blow had been received upon the occipital bone. Case XII. and XV. of Dr. Smith's are cases of "fracture from violence applied to back of neck." Taking these facts into consideration, to-wit, that violence had been applied to the back of the neck, or the posterior arch of the atlas; that the patient had a syphilitic history, and that on bending the neck a tingling sensation was transmitted from the posterior columns of the cord along the brachial plexus to the nerves of the upper extremities and their terminal filaments—taking these facts into consideration, that there was *some* lesion in the upper portion of the vertebral column, and I was led to localize that lesion either in the posterior border of the foramen magnum or in the posterior arch of the atlas, by finding that the disordered sensation occurred during the first stage of the act of nodding. What the nature of the lesion might be, I did not pretend to say, but consider it to be one of three things—fracture, periostitis, or caries.

February 22.—T. H. states that he caught a violent cold on the evening of the 20th, since which he has not felt the tingling, and cannot produce it by nodding. Tried in vain to do so in my presence. Attributes the cure to rest from his ordinary occupation.

The violent cold of which the man camplained was simply the effect of his medicine. It was a well-pronounced case of iodism.

I saw T. H. again on the 30th April, two months later. He had rested for several days before returning to his work, since which he had experienced no return of the symptoms that had formerly caused him so much anxiety.

Now, this favorable result of the case only serves to render the diagnosis still more difficult. If the tingling had been constant, I should attribute the disordered sensation to exhaustion of nerve force in the upper extremities; but in that case the symptom ought to be common among car-drivers, and T. H. stated that he had not known any of his fellow employès to suffer from it. It was not constant, however, being produced by bending the head forward; and I am forced to conclude that there was a slight degree of periostitis causing pressure on the posterior columns of the cord at its commencement, during a certain movement of the head. The patient was one of those persons, of whom we see so many, peculiarly susceptible to the action of iodide of potassium, and a few doses of the drug were sufficient to cause absorption of the slight inflammatory thickening which had taken place.—*Philadelphia Medical Times.*

